# Feeding practices of low-income mothers: how do they compare to current recommendations?

**DOI:** 10.1186/s12966-015-0179-3

**Published:** 2015-03-07

**Authors:** Thomas G Power, Sheryl O Hughes, L Suzanne Goodell, Susan L Johnson, J Andrea Jaramillo Duran, Kimberly Williams, Ashley D Beck, Leslie A Frankel

**Affiliations:** Washington State University, Pullman, WA USA; Children’s Nutrition Research Center, Baylor College of Medicine, 1100 Bates, Houston, TX 77030-2600 USA; North Carolina State University, Raleigh, NC USA; University of Colorado Denver, Aurora, CO USA; University of Houston, Houston, TX USA

**Keywords:** Mothers, Feeding behaviors, Obesity

## Abstract

**Background:**

Despite a growing consensus on the feeding practices associated with healthy eating patterns, few observational studies of maternal feeding practices with young children have been conducted, especially in low-income populations. The aim of this study was to provide such data on a low income sample to determine the degree to which observed maternal feeding practices compare with current recommendations.

**Methods:**

Eighty low-income mothers and their preschool children were videotaped at dinner in their homes. Mothers were chosen from a larger study to create a 2 X 2 X 2 design: maternal ethnicity (African American vs. Latina) by child gender by child weight status (healthy weight vs. overweight/obese). Observers coded videotapes for a range of maternal feeding strategies and other behaviors.

**Results:**

Many mothers spent considerable time encouraging eating—often in spite of the child’s insistence that he or she was finished. Mothers talked little about food characteristics, rarely referred to feelings of hunger and fullness, and made more attempts to enforce table manners than to teach eating skills. Latina mothers showed higher levels of teaching eating skills and encouraging their children to eat; African American mothers showed higher levels of enforcing table manners and getting children to clear their plates. Mothers of boys used more unelaborated commands and less questions/suggestions than mothers of girls. Finally, compared to mothers of overweight/obese children, mothers of healthy weight children showed higher levels of encouraging eating and lower levels of discouraging eating.

**Conclusions:**

Most of the mothers in this study did not engage in feeding practices that are consistent with current recommendations. They did this, despite the fact that they knew they were being observed. These results should be used to inform future research about the motivations behind mothers’ feeding practices and the development of interventions by helping identify areas in greatest need of change.

## Background

Overweight and obesity pose significant health problems for children and adolescents [1]. Childhood obesity rates have tripled in the past three decades [2]. These rates are even higher for low-income and minority children [3]. It is well recognized that parents play a fundamental role in shaping the trajectory of eating behaviors in children and thus the development of overweight [4]. Increasingly, parenting styles and practices have been associated with child intake and obesity [5]. Childhood obesity has been linked to both highly controlling and highly indulgent parenting in the eating and non-eating domains [4-6]. Researchers have suggested that these parenting practices can interfere with children’s self-regulation of caloric intake, therefore increasing their obesity risk [7].

Currently, researchers and practitioners advocate the use of responsive feeding practices to minimize the likelihood of childhood obesity [8-11]. Responsive feeding, during the preschool years, is characterized by caregiver guidance and recognition of the child’s cues of hunger and satiety. Nonresponsive feeding is characterized by a lack of reciprocity between parent and child, with the caregiver taking excessive control of the feeding situation (forcing, pressuring, or restricting food intake), the child controlling the feeding situation (indulgent feeding), or low levels of caregiver involvement (uninvolved feeding) [8-11]. Other feeding practices that researchers and practitioners encourage (mostly to increase child consumption of fruits and vegetables) include: 1) presenting novel foods frequently to encourage liking [12]; 2) encouraging interest in new foods through conversation and granting children opportunities to explore foods [13]; 3) enthusiastic modeling of healthy food consumption [14]; and 4) facilitating the development of independent eating skills and not overemphasizing table manners [15]. Based upon some of this literature, the American Academic of Pediatrics recommended that parents of young children: “provide a healthy array of foods in the correct portion size and allow children to decide what and how much to eat from what they are offered [16].”

Despite these recommendations, few observational studies have examined the degree to which parents adopt responsive feeding behaviors, especially in low-income, minority samples-populations at high risk for childhood obesity [3]. In a systematic review of responsive feeding and child weight published in 2011, most studies relied on parent-report measures [17]. Only four studies employed observational measures, and two were studies of mothers and infants [18,19]. Similarly, Lumeng and colleagues [20], in 2012, noted that “Since 1981, the few studies that have evaluated maternal feeding style by direct observation in association with child weight status have included only ~200 child participants and > 80% of these participants have been white” (p. 640). The reliance on questionnaires is problematic for several reasons—social desirability, under-reporting of negative interactions, parents’ limited awareness of their own behavior, and problems in recall [21-25]. Haycraft and Blissett [26], for example, found that mothers’ reports of their own feeding practices showed no significant correlations with observed feeding behavior (although some correlations for fathers were significant).

Given than child obesity rates differ as a function of child ethnicity and gender [1,3], a second issue concerns differences in maternal feeding practices as a function of maternal ethnicity, child gender, and child weight status. Previous research on these three variables is limited and inconsistent. Very limited data on ethnic differences are available, for example, because the vast majority of observational studies of feeding practices have examined white, middle class mothers. In one exception, an observational study of mothers of preschool children feeding their children a snack, Lumeng and colleauges [20] found that non-Hispanic, white mothers used fewer assertive and intrusive feeding prompts than ethnic minority mothers. No other studies of ethnic differences in observed maternal feeding behavior were found. In two self-report studies of maternal feeding practices, Hughes and colleagues [27,28] found that a greater proportion of low-income Latina mothers showed an indulgent feeding style (high responsiveness, low demandingness) compared to mothers from other ethnic groups.

Several observational studies have examined how maternal feeding behavior varies as a function of child gender. In three studies of young children (ages 3-8), mothers of boys encouraged their children to eat more frequently than mothers of girls [29-31]. Similarly, an observational study of 7-13 year olds [32] showed that parents of boys exerted more behavioral control during mealtime than parents of girls. However, one large study that included ethnic minority children [20] found no child gender differences in maternal feeding behaviors.

Research on the effects of child weight status on parental feeding behavior is inconsistent. Two observational studies by Klesges and colleagues found that parents of obese children encouraged their children to eat more frequently than parents of healthy weight children [33,34], whereas other observational studies found no relationship between eating prompts and child weight status [20,35,36]. Interestingly, child and parent self-report studies conclude that parents of obese children report lower levels of pressure to eat [37,38]. Observational studies of the *quality* of parental control over child eating show that parents of obese children show more assertive, intrusive, authoritarian, or permissive control [20,32]. Finally, Birch and colleagues [35] found that mothers of thinner children (as assessed with skinfold thickness) talked with their children more about nonfood topics during lunch in a laboratory session than mothers of children with higher fat levels.

Without observational data on feeding in low-income families, it is difficult to determine whether or not maternal feeding behaviors might contribute to obesity risk in low-income families, and if so, to determine which practices might best be targeted in education and prevention. The major purpose of this study was to examine the degree to which low-income, ethnic minority parents show feeding practices that are consistent with current recommendations for responsive feeding. The second purpose was to examine differences in maternal feeding behavior as a function of maternal ethnicity, child gender, and child weight status. This was accomplished through direct observations of feeding in a sample of low-income African American and Latina mothers and their preschoolers.

## Methods

### Participants

The videotapes coded for this study came from a larger study of parent-child interaction at dinner [39]. In this larger study of 177 families, observers coded parent-child interactions at three separate meals per family—the results of this live coding are reported in Hughes et al. [39]. These observations had been videotaped for later coding and analysis. To examine differences in maternal behavior as a function of maternal ethnicity, child gender, and child weight status, a subset of the videotapes was coded for the present paper.

Videotapes of eighty mothers and their preschool children were selected. Mothers were chosen from the larger sample to create eight groups (with 10 mothers per group) making up each of the cells of a 2 X 2 X 2 design: maternal ethnicity (African-American vs. Latina) X child gender X child weight status (healthy weight vs. overweight or obese). A cell size of 10 was chosen because the smallest cell was made up of 10 African American mothers of overweight/obese female children. Therefore, 10 mothers were randomly chosen from each of the remaining seven cells. The number of participants in the cells we selected from ranged from 16 to 26 for mothers of healthy weight children and from 10 to 17 for mothers of overweight/obese children. Thirty nine percent of the children in the larger study were classified as overweight or obese. Eighty mother-child pairs was a sufficiently large sample size to detect a medium effect size (*f* = .25) with a power value of .89 [40].

Sample demographics are presented in Table 1. Children and adults were classified as healthy weight or overweight/obese based upon CDC criteria [41,42]. The second meal was chosen for coding because we expected more reactivity in the first observation and not all families were observed for a third meal.

**Table 1:** **Characteristics of the Latina and African American mothers of preschoolers in Houston, Texas (** ***n*** **= 80)**

	**Frequencies**
Ethnicity	
Latina	40
African-American	40
Education of Mother	
Less than High School Diploma	21
High School Diploma	19
Some College	32
College Graduate	6
Missing Data	2
Marital Status of Mother	
Married	31
Divorced	1
Separated	13
Never Married	27
Missing	8
Employment of Mother	
Employed Part-time	24
Employed Full-time	20
Not Employed	36
Child Gender	
Female	40
Male	40
Child BMI z score, Mean (SD)	1.02 (1.06)
Child Weight Status	
Healthy Weight (>5^th^ to < 85^th^BMI percentile)	40
Overweight/Obese (BMI ≥ 85^th^ percentile)	40
Mother BMI, Mean (SD)	31.12 (8.36)
Mother Weight Status	
Healthy Weight (18.5 kg/m^2^ < BMI < 25 kg/m^2^)	17
Overweight/Obese (BMI ≥ 25 kg/m^2^)	63
Age, Mean in Years (SD)	
Parent	32.31 (7.49)
Child	4.51 (0.64)

### Procedures

As discussed in Hughes et al. [39], mothers were recruited through Head Start Centers and consented before participating in the study. All mothers were low income because as Head Start participants, families are required to be at or below the federal poverty level. Parents received an incentive (graduated in amount) at the end of each of the three observations. Parents were told to do what they normally do at dinner time and to feed their child as they usually do. Two cameras were placed in the room where the family planned to eat. One camera was directed so that the mother’s face could be recorded and the other camera was directed so that mother/child interactions were in view. Cameras were turned on after the family sat down and food was served. Two live coders were also present during the meal while the cameras were recording the parent-child interactions. Although most observations took place at a kitchen or dining room table, five children were observed while eating on a couch or a chair in the living room. In all but eight cases, the mothers sat down and ate the meal with their child. Clearly the families were aware of the cameras; however, after a few minutes the families appeared to no longer pay attention to them. During nine observations, fathers were also present, but only maternal behavior was coded for this study. The study was reviewed and approved by the Institutional Review Board at Baylor College of Medicine.

### Videotape coding

All videotapes were transcribed in the language used by the mother and child (32 of the videotapes were in Spanish). Using the Noldus Observer software (Observer XT, Noldus Information Technology, Wageningen, Netherlands), videotapes were coded by four B.A. level employees (three were bilingual) blind to the purposes of the study. One quarter of the videotapes were coded independently by a second bilingual observer to assess inter-observer agreement. The coders were unaware of which observations had been selected for reliability assessment. Agreement was assessed with Cohen’s kappa [43].

**Figure 1:**
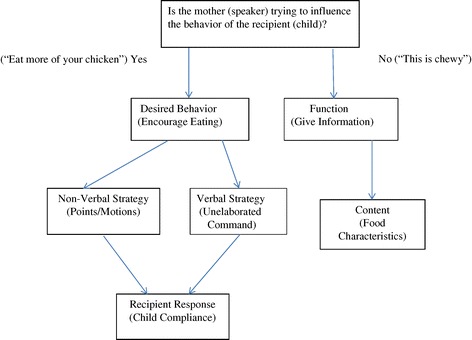
**Flow chart representing videotape coding process.**

Employing event coding, all maternal and child verbalizations, along with a number of nonverbal behaviors, were coded with a system adapted from Baumrind and Black [44] and Cousins, Power, and Olvera [45]. The codes were mutually exclusive and exhaustive. They were developed by expanding on the systems used in these previous studies through examination and discussion of pilot videotapes. Data from only the maternal verbalizations and nonverbal behaviors are presented in the current paper (child verbalizations were not analyzed). As illustrated in Figure 1, all maternal attempts to influence child behavior and child attempts to influence maternal behavior were coded, along with all other verbalizations between mother and child. The maternal behaviors coded included positive strategies representative of responsive feeding practices, as well as controlling feeding strategies.

The maternal behaviors chosen to assess responsive feeding practices in this study were: maternal references to internal hunger and fullness cues; discussion of foods and their characteristics; instruction in independent eating skills; enthusiastic modeling; and the use of non-directive, facilitating strategies to influence child eating (e.g., suggestions, questions, reasoning, helping) rather than more forceful, intrusive strategies (e.g., unelaborated commands, forces eating, spoon feeding). Nonresponsive feeding included frequent prompts to eat, forceful strategies, or a focus on table manners rather than teaching eating skills.

The variables and codes involving influence attempts analyzed for the current paper are listed in the following section. Kappa statistics for the various aspects of the coding system ranged from .72 to .86 with a mean of .77.

### Codes analyzed for current paper

I. Total Frequency of Maternal Attempts to Influence Child Behavior During Meal
II. Child Behaviors Mothers were Trying to Influence (i.e., Desired Behaviors)
  A. Encourage Eating
  B. Encourage Child to Eat All the Food on the Plate
  C. Encourage Child to Eat a Different Food
  D. Discourage Eating
  E. Enforce/Teach Table Manners
  F. Teach Eating Skills
  G. Internal Cues Reference to Encourage Eating
  H. Internal Cues Reference to Discourage Eating
  I. Other Food Related Behaviors (e.g., pass food, help sibling serve food)
  J. Non Food-Related Behaviors (e.g., discourage TV watching, be nice to sibling)
III. Maternal Verbal Strategies used to Influence Child Behavior
  A. Hint/Acknowledge
  B. Enthusiastic Modeling
  C. Question/Suggestion
  D. Praise
  E. Reason/Instruct
  F. Unelaborated Command
  G. Verbal Pressure (e.g., “You *have* to eat it”)
  H. Disapprove/Scold
  I. Promise Food Rewards
  J. Threaten Food Punishments
  K. Promise Non-Food Rewards
  L. Threaten Non-Food Punishments
IV. Maternal Non-Verbal Strategies to Influence Child Behavior
  A. Moves Self Closer
  B. Moves Something Closer
  C. Points/Motions
  D. Helps
  E. Spoon Feeds
  F. Physically Forces
V. Total Frequency of Maternal Non-Influence Attempts
VI. Types of Non-Influence Attempts
  A. References to Food Characteristics (e.g., appearance, smell, preparation)
  B. References to Other Food-Related Content (e.g., food placement, utensils)
  C. References to Target Child
  D. References to Mother
  E. References to Other People
  F. References to Non-Food Related Content (e.g., “It’s going to rain tomorrow.”)
  G. Clarification

### Data analysis

The variables for the analyses were the total frequency of influence and non-influence attempts, along with a number of proportions corresponding to the specific coding categories. Proportions were used for the main analyses because the total number of influence and non-influence attempts varied widely across families. For the influence attempts, separate proportions were calculated for the desired behavior categories, the verbal strategies, and the nonverbal strategies. The numerators for these proportions were the frequencies that a particular code occurred and the dominator for each was the total frequency of influence attempts (verbal plus nonverbal). For example, if a mother encouraged her child to eat 10 times during the observation and the total number of influence attempts was 50, the proportion for that mother was 0.20.

To examine ethnic, child gender, and child weight status differences in maternal behavior, five separate 2 X 2 X 2 MANOVAs (maternal ethnicity X child gender X child weight status) were run. The dependent variables for these five analyses were: 1) the frequencies of influence and non-influence attempts; 2) the proportion measures for eight of the desired behavior codes (the two internal cues references codes were not included due to low frequency of occurrence); 3) the proportion measures for six of the verbal strategy codes (praise, discourage/scold, and the four reward and punishment codes were not included due to low frequency of occurrence); 4) the proportion measures for the six nonverbal strategies; and 5) the proportion measures for the seven non-influence attempts codes. Approximate F statistics were calculated using Wilk’s lamda. To help protect against Type I error, univariate effects (ANOVAs) were only examined if the corresponding multivariate effect was significant (*p* < .05).

## Results

Examination of the frequency distributions of the influence and non-influence attempts revealed one extreme outlier—one mother engaged in 200 influence attempts and 117 non-influence attempts during the dinnertime observation. This mother-child pair was dropped because her values were greater than four standard deviations above the mean. After dropping this observation, across families, the mean number of maternal influence attempts per observation was 34.2 and the mean number of non-influence attempts was 13.1 (see Table 2).

**Table 2:** **Frequency of feeding behaviors of Latina and African American mothers of preschoolers during mealtime observations**

**Codes**	**Percent Non-Zero**	**Mean**	**SD**	**Range**
**Frequency of Influence Attempts**	99	34.2	29.6	0-132
**Desired Behaviors (proportion of total influence attempts)**				
Encourage Eating	99	.42	.19	0-.90
Eat All	44	.03	.05	0-.21
Eat Different Food	45	.06	.11	0-.58
Discourage Eating	78	.09	.11	0-.67
Table Manners	76	.12	.11	0-.35
Eating Skills	60	.09	.12	0-.54
Internal Cues Encourage Eating	13	.01	.02	0-.13
Internal Cues Discourage Eating	18	.01	.02	0-.13
Other Food	70	.10	.11	0-.50
Non-Food	53	.07	.14	0-1.00
**Verbal Strategies (proportion of total influence attempts)**				
Hint/Acknowledge	62	.05	.06	0-.28
Enthusiastic Modeling	36	.02	.03	0-.15
Question/Suggest	95	.25	.16	0-.71
Praise	27	.01	.04	0-.27
Reason/Instruct	65	.07	.09	0-.50
Unelaborated Commands	99	.54	.18	0-1.00
Verbal Pressure	44	.02	.03	0-.14
Discourage/Scold	14	.01	.02	0-.14
Food Rewards	12	.004	.01	0-.09
Food Punishments	9	.003	.01	0-.07
Non-Food Rewards	6	.001	.01	0-.04
Non-Food Punishments	22	.01	.03	0-.18
**Non-Verbal Strategies (proportion of total influence attempts)**				
Moves Self Closer	44	.04	.07	0-.37
Moves Something Closer	49	.04	.06	0-.33
Points/Motions	76	.11	.12	0-.75
Helps	45	.03	.05	0-.19
Spoon Feeds	22	.02	.05	0-.32
Physically Forces	37	.02	.04	0-.19
**Frequency of Non-Influence Attempts**	96	13.1	16.1	0-81
**Content of Non-Influence Attempts (proportion of total non-influence attempts)**				
Food Characteristics	46	.09	.12	0-.50
Other Food Content	64	.34	.31	0-1.00
References to Child	80	.32	.29	0-1.00
References to Mother	28	.03	.07	0-.50
References to Other People	32	.05	.10	0-.50
Non-Food References	32	.06	.13	0-.88
Clarification	54	.11	.18	0-1.00

Meals ranged in duration from seven to forty minutes, with a mean of 18.1 minutes (SD = 7.4). As expected, the frequency of influence attempts, *r*(77) = .39, *p* < .001, and non-influence attempts, *r*(77) = .32, *p* < .001, were positively correlated with the length of the meals.

### Descriptive analyses

#### Influence attempts: desired behavior

Table 2 presents for each code, the percent of mothers who showed that behavior at least once (“percent non-zero”), along with the sample means, standard deviations, and ranges for the measures. The most common desired behavior by far was “encourage eating.” This occurred at a mean rate over three times higher than the next desired behavior—“table manners.” Other desired behaviors occurring less frequently were “teach the child eating skills,” “discourage eating,” and “other food-related” desired behaviors. A closer examination of the behaviors making up the discouraging eating category (data not presented in the table) showed that about 60% of these came toward the end of the meal when the parent was trying to get the child to finish the meal and stop eating. Requests for the child to eat all of the food on the plate and references to internal cues were uncommon.

#### Influence attempts: verbal strategies

Table 2 shows that by far, the most common verbal strategy was “unelaborated commands,” which occurred about twice as frequently as the next most common strategy, “question/suggestions.” The third most common strategy was “reason/instruct” followed by “hint/acknowledge.” All of the remaining strategies were very low in occurrence, including bribes and threats, praise and scolding, and enthusiastic modeling.

#### Influence attempts: nonverbal strategies

As shown in Table 2, “points/motions” was by far the most common nonverbal strategy. The next two most common strategies were “moves self closer” and “moves something closer,” followed by “helps,” “spoon feeds,” and “physically forces” which all occurred infrequently. The actual use of rewards and punishments was extremely low—bribes and threats were more common, but as mentioned above, occurred infrequently.

#### Non-influence attempts: content

The most common content that mothers referred to in their non-influence attempts was the target child. For example, a mother might say something such as “What did you do in school today?” As shown Table 2, mothers referred to the target child more often than they referred to other people or to themselves. Mothers frequently made references to food, but rarely to “food characteristics.”

### Differences in maternal behavior as a function of maternal ethnicity, child gender, and child weight status

The multivariate effect of maternal ethnicity only was significant for the desired behavior codes, F(8, 63) = 5.26, p < .001, eta^2^ = .40. As shown in Table 3, examination of the univariate effects showed that Latina mothers scored higher on “encourage eating,” “eat a different food,” and “teach eating skills,” whereas African American mothers scored higher on “eat all food,” “discourage eating,” “table manners,” and “other food” desired behaviors. There was no significant ethnic difference in non-food influence attempts.

**Table 3:** **Ethnic differences in desired behaviors** ^**a**^

**Codes**	**African American M (SD)**	**Latina M (SD)**	***F*** **(1, 70)**	***p*** ≤	**eta** ^**2**^
Encourage Eating	.38 (.19)	.47 (.17)	4.68	.05	.06
Eat All	.04 (.06)	.02 (.04)	2.86	.10	.04
Eat Different Food	.03 (.06)	.09 (.14)	5.52	.05	.07
Discourage Eating	.13 (.13)	.05 (.07)	10.97	.001	.14
Table Manners	.14 (.11)	.09 (.10)	3.88	.05	.05
Eating Skills	.06 (.08)	.12 (.14)	5.18	.03	.07
Other Food	.13 (.11)	.07 (.10)	5.59	.02	.07
Non-Food	.07 (.09)	.08 (.18)	.10	ns	.001

^a^MANOVA significant – see text.

The multivariate effect for child gender only was significant for verbal strategies, F(6,65) = 2.57, p < .05, eta^2^ = .19. This was due to two univariate differences: mothers used more unelaborated commands with their boys (*M* = .61, *SD =* .18) than their girls (*M* = .47, *SD* = .16), *F*(1, 70) = 14.48, *p* < .001, eta^2^ = .17, and more questions/suggestions with their girls (*M* = .30, *SD* = .17) than their boys (*M* = .20, *SD* = .14), *F*(1, 70) = 7.04, *p* < .01, eta^2^ = .09.

There were three significant multivariate effects involving child weight status: the child weight status main effect was significant for the frequency of influence/non-influence attempts, *F*(2,70) = 3.19, p < .05, eta^2^ = .08, and for the desired behavior codes, *F*(8,63) = 2.80, *p* = .01, eta^2^ = .26. The child weight status by ethnicity interaction, *F*(2,70) = 5.98, *p* < .01, eta^2^ = .15, was significant for the frequency of influence and non-influence attempts.

Univariate analyses showed that mothers of healthy weight children engaged in more total influence attempts during the meal (*M* = 40.82, *SD* = 32.50) than mothers of overweight or obese children (*M* = 27.72, *SD* = 25.30), *F*(1,71) = 3.80, *p* = .05, eta^2^ = .05. Mothers of healthy weight children showed higher levels of encourage eating (*M* = .48, *SD* = .19) than mothers of overweight/obese children (*M* = .37, *SD* = .17), *F*(1,70) = 8.77, *p* < .01, eta^2^ = .11. Mothers of overweight/obese children, in contrast, showed higher levels of discourage eating (*M* = .12, *SD* = .14) than mothers healthy weight children (*M* = .06, *SD* = .06), *F*(1,70) = 8.53, *p* < .01, eta^2^ = .11.

Finally, the univariate interaction between maternal ethnicity and child weight status was significant for the total number of non-influence attempts, *F*(1,71) = 4.79, *p* < .05, eta^2^ = .06. For Latina mothers, mothers of healthy weight children engaged in more total non-influence attempts (*M* = 16.84, *SD* = 19.88) than mothers of overweight or obese children (*M* = 8.50, *SD* = 9.36). In contrast, the opposite was true for African American mothers with mothers of healthy weight children engaging in fewer total non-influence attempts (*M* = 9.70, *SD* = 6.97) compared to mothers of overweight or obese children (*M* = 17.40, *SD* = 21.97).

There were no significant multivariate effects for the nonverbal strategies or the non-influence attempt content codes.

## Discussion

Together, these observational analyses show that many of the mothers in this sample did not employ feeding practices consistent with current recommendations for the feeding of young children [8-11], including the recommendations by the American Academy of Pediatrics [16]. Rather than providing children with food and then allowing them to decide what and how much to eat, a significant portion of the mothers in this sample spent considerable time encouraging their children to eat—often in spite of their insistence that they were finished (in data not presented here, 84% of the children indicated that they wanted to stop eating at some point during the mealtime observations—often multiple times). Mothers talked little about the food and its characteristics, rarely referred to feelings of hunger and fullness, and focused more on table manners than on teaching children eating skills. In trying to influence child behavior, mothers relied primarily on unelaborated commands and rarely used instruction, helping, reasoning, or praise. Overall, the focus of maternal behavior seemed more on ensuring that the child ate enough food and that the child exhibited proper behavior.

The degree to which mothers encouraged children to eat varied widely across the sample. Combining the frequencies of all of the encouraging eating codes (i.e., encourage eating, eat all, eat different food, and internal cues—encourage eating), the mean number of eating prompts was about 16 occurrences per mealtime observation—about half of all influence attempts observed. Examination of the frequency distributions showed that about one third of the mothers engaged in 16 or more eating prompts (the highest number was 91) (an authoritative or authoritarian feeding style), whereas about one quarter of the mothers engaged in 5 attempts or less (an indulgent or uninvolved style). Discouraging eating was not common—the mean occurrence was about three per dinnertime observation—less than nine percent of all influence attempts.

Several explanations can be offered for why many mothers encouraged eating even after the children indicated that they were done. First, for low income mothers, food security is often an issue [46]. When mothers are in a position to provide their child with a good meal, they may encourage their children to eat, even if the child has indicated that he or she is finished. Second, mothers may believe that it is important that their children consume enough food to meet their daily energy requirements and they may feel that they themselves are in a better position than their child to know when the child has eaten enough. Finally, mothers may encourage child eating at meals to save time, to prevent having to feed a hungry child later, or to ensure that the child does not go to bed hungry.

Examination of the data on non-influence attempts showed that mothers rarely commented on food characteristics—instead they focused primarily on the child’s behavior and other food-related topics, such as “Do you want me to put some sauce on it?” By not commenting on food characteristics (getting the child to think and talk about the food’s taste, texture, appearance, etc.), these mothers were missing opportunities to encourage children’s interest in trying and developing preferences for new foods [13]. The same is true of maternal references to internal hunger or fullness cues. Only about a quarter of the mothers made any references to these internal cues, and among those who did, the vast majority made only one (or sometimes two) per observation (usually at the end of the meal to check to make sure that the child had eaten enough). By frequently encouraging children to eat without making references to these internal cues, mothers may be teaching children to ignore their internal cues of fullness, thereby interfering with the self-regulation of caloric intake [47].

Besides encouraging children to eat, most other maternal influence attempts focused on enforcing table manners. Influence attempts involving manners were common (about 75% of mothers enforced such rules). This is consistent with previous research on the importance of obedience to authority in low-income samples [48-50]. In addition to manners, about 60% of the mothers spent some time teaching eating skills. However, the use of helping and instruction was rare—these mothers relied mostly on unelaborated commands (e.g., “Be careful—don’t spill your milk”). Again, the focus on encouraging proper behavior to the exclusion of encouraging independent eating skills is inconsistent with current recommendations.

Examination of *how* mothers tried to influence children’s behavior showed that the only common strategies besides direct commands (about half of all maternal influence attempts) were questions and suggestions (about one quarter of all influence attempts). The high use of direct commands is consistent with other studies of low-income mothers [31,45]. Given that authoritative parenting has been shown to be a protective factor against the development of childhood obesity [4], increasing the use of reasoning, instruction, and praise are teachable skills that could be included in interventions related to parenting and feeding. Moreover, because less power-assertive methods of parental control ultimately elicit better child cooperation by allowing children greater autonomy and giving them the sense that they are involved in a reciprocal relationship [51,52], the use of less directive strategies such as questions and suggestions may be more successful in having a long-term impact on child eating behavior.

Interestingly, several feeding strategies that have received considerable attention in the literature-enthusiastic modeling [14], telling children to clean their plates [53], and using food as a reward [54] were each low frequency, accounting for a very small portion of influence attempts. Only about one third of the mothers made a positive comment about the taste of the food (enthusiastic modeling) or told the child to clean his or her plate, and only 12% of mothers promised food as a reward. So despite the numerous experimental studies that show that these strategies *can* affect children’s self-regulation of intake and the development of food preferences, they may not be the strategies that actually *do* affect these outcomes in low income populations, given their low frequency of occurrence [55] (most of the experimental studies were conducted with middle class children). This suggests that experimental research on the effectiveness of less directive and more common feeding strategies during mealtimes may be worthwhile.

The only type of modeling examined in this study was “enthusiastic modeling,” where mothers paired the eating of a desired food with a positive comment about it. It is likely that other types modeling not studied here (e.g., simply eating a desired food in the child’s presence) are effective ways to influence child eating behavior [56]. Moreover, the use of food as a reward might occur more frequently in other situations outside of meals (e.g., motivating child behavior through snacks or treats between meals).

The differences we observed in maternal feeding behavior help replicate and/or extend previous work in this area. The greatest number of differences were for maternal ethnicity. Latina mothers showed higher levels of encouraging eating, getting their child to eat a different food, and encouraging the development of eating skills. African American mothers showed higher levels of discouraging eating, trying to get their children to eat all of the food on their plate, enforcing table manners, and trying to influence “other food-related” behaviors. Despite these ethnic differences in encouraging and discouraging eating, studies on BMI in young children show no significant differences in overweight and obesity rates between African American and Latino preschool children [1,3]. Therefore, the ethnic differences in feeding style identified here may not lead to differences in weight status across the groups (possibly due to ethnic differences in the food served). Similarly, the difference in encouraging eating a different food might be a function of differences in the foods served by mothers of the two ethnicities. This study, to our knowledge, is the first study to examine such ethnic differences in observed feeding behaviors, so it is important to replicate these findings in other samples, as well as examine how feeding behaviors interact with foods served in increasing or decreasing obesity risk.

The findings of this study are inconsistent with three previous studies that found that parents of boys encouraged eating more often in their boys than in their girls [29-31]. However, the finding that mothers of boys used more commands and mothers of girls used more questions/suggestions, is consistent with a study of older children that found that parents exerted more control over their boys than girls during mealtime [32]. A national study [3] showed that for both African American and Latina/o preschoolers, obesity rates were higher for boys than for girls. This difference may be due, in part, to parents’ tendency to use more forceful strategies during mealtime with their boys than with their girls—thus overriding children’s responsiveness to their internal cues of fullness.

Finally, the differences in child weight status are consistent with several self-report studies that show that parents of healthy weight children pressure their children to eat more frequently than parents of obese children [37,38]. This could be due to mothers of thinner “picky” eaters trying to increase child consumption, to mothers of overweight or obese children not having to pressure eating because their children eat on their own, or to mothers of overweight or obese children not pressuring eating because they are concerned about the child’s weight status (or some combination of all three). The results are inconsistent with two smaller-scale studies by Klesges and colleagues who found a positive relationship between child obesity and the frequency of maternal eating prompts [33,34]. The results are also inconsistent with the hypothesis that children are at increased risk for obesity if their parents are highly controlling at mealtime (thereby decreasing children’s responsiveness to internal cues of fullness). One possible reason for the studies that find no relationship between child weight status and observed maternal eating prompts [20,35,36] is that in the short run, parents may use high pressure tactics with healthy weight children who are “picky eaters,” but in the long run, these high pressure practices—at least for some children—may lead to less child responsiveness to internal cues of fullness and subsequent increased obesity risk. In cross sectional studies such as those reviewed above, the operation of these two conflicting factors might cancel one another out and lead to no correlation between maternal eating prompts and child weight status. Clearly, the relationship between weight status and maternal feeding behavior is complex, and longitudinal research needs to be conducted to further understand these relationships—especially in light of a recent study by Rhee and colleagues who found, in a maternal self-report study, that controlling feeding practices become more common after, not before child weight gain [57].

Although these observational data provide a wealth of information not currently available on the feeding practices of low-income, minority mothers, they should be viewed within the limitations of the study. Only low-income, African-American and Latino mothers whose children were enrolled in Head Start participated. This sampling strategy excluded mothers of other social classes and ethnicities, along with mothers whose children did not participate in center-based childcare (e.g., stay at home mothers, children in family-based care). The fact that so few fathers were present also raises some concerns about representativeness, and future observational studies should investigate more directly the role of fathers e.g., [26].

Observational methods have their limitations as well [58,59]. First, observations of parent-child interactions do not provide insight into the inner thoughts of those observed. Second, parents and children may alter their behaviors just by the nature of being observed, often in a socially desirable direction. Third, observational methods may capture only a small snippet of interactions in time such as family dinners in the home. For example, most observations do not capture interactions in restaurants or fast food outlets and eating while watching TV or in automobiles. Furthermore, low frequency behaviors such as yelling at or punishing children are not usually captured through observations thus limiting the ability to generalize observations to the entire range of behaviors that parents practice. Despite these limitations, observations still comprise an optimal way to measure *how* parents feed their children (using positive versus negative affect when delivering messages) and *what* they are doing during eating occasions (e.g. asking questions, giving hints, delivering direct commands). Moreover, observational measures have repeatedly been shown to assess important individual differences in parent-child interaction that are powerful predictors of child development outcomes [21,22].

To address the issue of reactivity, future studies should increase the number of days that videotapes are made, and possibly have parents operate the video camera with no observers present [59]. To explore maternal motivations for adopting these practices, or the barriers that threaten adoption of more responsive feeding practices, future studies should combine observational with self-report methods (e.g., interviews, questionnaires, focus groups) to address these issues. Finally, given the bi-directional nature of mother-child interactions [60-62], future studies and analyses should examine the bi-directional nature of the feeding process.

## Conclusions

These observational findings provide a detailed picture of mother-child interaction during mealtime in low-income families. They help identify areas that could be addressed in helping mothers engage in feeding practices that might reduce their children’s obesity risk. These include: increasing maternal sensitivity and responsiveness to children’s satiety cues; addressing over-controlling and indulgent feeding patterns; giving mothers strategies for focusing as much on the teaching of independent eating skills as they do on manners and etiquette; and encouraging greater mother-child conversation about food characteristics. Interventions to do this, however, need to be sensitive to the larger sociocultural context in which these practices occur [59,63].

Future research should examine these feeding practices in other populations that vary in ethnicity (e.g., European American and Asian American parents), education, and social class. Moreover, longitudinal research would help to examine the degree to which the patterns shown here, combined with data on children’s diet and activity level, predict the development of childhood obesity. Given that most studies of responsive feeding have been conducted with middle-class, European American mothers, it is possible that different relationships with child health outcomes may emerge in lower income samples. Such information would be useful in helping develop programs that might facilitate healthy eating patterns and promote child health.
